# Opportunities and Challenges of Multi-Ion, Dual-Ion and Single-Ion Intercalation in Phosphate-Based Polyanionic Cathodes for Zinc-Ion Batteries

**DOI:** 10.3390/molecules29204929

**Published:** 2024-10-18

**Authors:** Lei Cao, Tao Du, Hao Wang, Zhen-Yu Cheng, Yi-Song Wang, Li-Feng Zhou

**Affiliations:** 1 State Environmental Protection Key Laboratory of Eco-Industry, School of Metallurgy, Northeastern University, Shenyang 110819, China; 2371880@stu.neu.edu.cn (L.C.); 2201664@stu.neu.edu.cn (H.W.); 2310690@stu.neu.edu.cn (Z.-Y.C.); wangys@smm.neu.edu.cn (Y.-S.W.); 2 Engineering Research Center of Frontier Technologies for Low-Carbon Steelmaking (Ministry of Education), School of Metallurgy, Northeastern University, Shenyang 110819, China

**Keywords:** phosphate-based polyanionic cathode, intercalation/deintercalation mechanisms, zinc-ion batteries

## Abstract

**Abstract:** With the continuous development of science and technology, battery storage systems for clean energy have become crucial for global economic transformation. Among various rechargeable batteries, lithium-ion batteries are widely used, but face issues like limited resources, high costs, and safety concerns. In contrast, zinc-ion batteries, as a complement to lithium-ion batteries, are drawing increasing attention. In the exploration of zinc-ion batteries, especially of phosphate-based cathodes, the battery action mechanism has a profound impact on the battery performance. In this paper, we first review the interaction mechanism of multi-ion, dual-ion, and single-ion water zinc batteries. Then, the impact of the above mechanisms on battery performance was discussed. Finally, the application prospects of the effective use of multi-ion, dual-ion, and single-ion intercalation technology in zinc-ion batteries is reviewed, which has significance for guiding the development of rechargeable water zinc-ion batteries in the future.

## 1. Introduction

Energy and the environment are two major issues that must be addressed for the survival of humanity and the development of society today [1]. The continuous depletion of fossil fuels has made energy shortages a significant problem. Thus, there is an urgent need to develop and utilize new types of energy sources [2]. Electrochemical energy sources with high energy and high power have attracted people’s attention [3]. Battery storage systems powered by clean energy are crucial for the global economic transition related to carbon neutrality and carbon peaking [4]. Rechargeable batteries have broad application prospects in electric vehicles, mobile devices, and renewable energy sources [5]. The future of the renewable energy integrated grid system requires low-cost, high-safety, and long-cycle-life rechargeable batteries [6]. Among the many rechargeable batteries, lithium-ion batteries are more widely used; however, the problems of limited lithium-ion battery resources [7], high cost, and poor safety [8] have attracted increasing attention. Therefore, finding a new element to supplement lithium-ion batteries has become an urgent issue. Table 1 shows that the atomic radius of zinc is similar to that of lithium. Additionally, zinc has excellent electrochemical properties, with a lower electrode potential and a larger volumetric capacity, making it an attractive option for new types of batteries. Consequently, zinc has gradually received attention. In recent years, zinc-ion batteries have gained traction due to their abundant reserves, low cost, low toxicity, and low electrode potential [9].

Aqueous rechargeable batteries use aqueous electrolytes [10]. Compared to conventional lithium-ion and lead-acid batteries, aqueous rechargeable batteries have unique advantages [11]. The aqueous electrolytes used in these batteries have a lower risk of combustion and higher thermal stability than organic solution electrolytes, enhancing battery safety [12]. Their high energy density allows them to store more energy and provide longer operating times [13]. Among the many cathode materials, polyanionic materials are categorized into silicates, phosphates, pyrophosphates, sulfates, and hybrid polyanionic compounds based on the type of anionic groups [14]. Polyanionic compounds have the general formula $A_{x}B_{y}(MO_{n}{)}_{z}$, where A stands for alkaline elements (Li, Na, K), B stands for metals (Mn, Ni, Cu, Co, or Zn), and M stands for P, S, Si, Mo, W. These compounds usually consist of $BO_{6}$ octahedra and $MO_{4}$ tetrahedra sharing oxygen atoms [15]. Compared to transition metal oxides, polyanionic compounds have gained research interest due to their multidimensional ionic diffusion channels, high structural stability during charging and discharging, and tunable intercalation/deintercalation platforms [16]. Currently, the electrodes available in aqueous rechargeable batteries are mainly phosphate-based polyanionic compounds [9], such as Na_3_V_2_(PO_4_)_3_ [17], VOPO_4_ [18], and so on, which are composed of aqueous systems with zinc-ion batteries. Compared with other materials, phosphate-based polyanionic cathode materials have unique advantages. For one thing, because of the limitation of water splitting potential, most cathodes could be incompatible with an aqueous rechargeable battery system. By introducing polyanionic groups into a transition metal oxide framework (i.e., inductive effect), the operation potential can be adjusted owing to the bonding strength and covalency of metal−oxygen (M-O). On the other hand. phosphate-based polyanionic cathode materials show high stability again moisture compared to layered oxide cathodes, which boosts the chemical stability of materials. Zinc-ion batteries have received widespread attention in recent years. Analyzing these batteries can explore the performance of cathode materials, determine the mechanism of zinc-ion batteries, and contribute to improving their efficiency.

**Figure 1:**
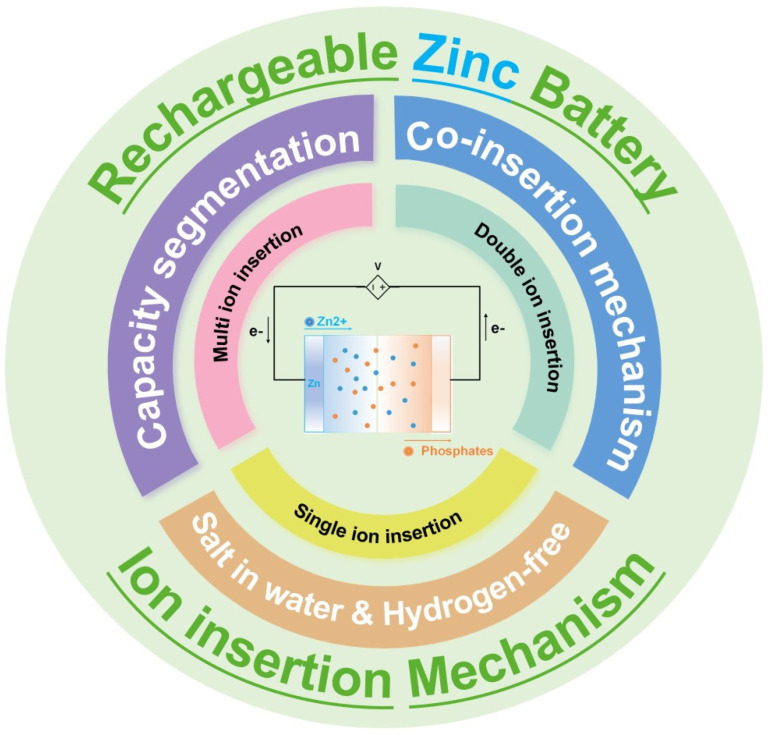
Schematic representation of multiple ion insertion mechanisms in rechargeable zinc phosphate-based batteries.

Multi-ion insertion is a key feature of batteries that can enhance their performance and application areas [19]. The joint action of multiple ions accelerates charge transfer between the positive and negative electrodes, resulting in a higher energy density and faster charge and discharge rates. However, multi-ion insertion also presents challenges, such as side reactions that affect battery performance and structural changes within the battery. Meanwhile, dual-ion insertion and single-ion insertion also have their special advantages and disadvantages. Therefore, a comparative discussion of the insertion of these three types of ions for can help us clarify the ion mechanism of the battery. To facilitate the discussion, these mechanisms are classified in this paper by dividing the action mechanisms of rechargeable zinc batteries into three categories: multi-ion insertion mechanism, dual-ion insertion mechanism, and single-ion insertion mechanism. Under these mechanisms, we discuss the capacity division among multiple ions, the hybrid insertion mechanism of dual ions, and the high electrolyte concentration and H+-free action of single ions. The main relationships are shown in Figure 1, The innermost layer of Figure 1 is a reaction schematic of zinc-ion batteries, and the first layer of circles is the insertion mechanisms of zinc-ion batteries, categorized in this paper as multi-ion insertion mechanism, dual-ion insertion mechanism, and single-ion insertion mechanism. The second layer are the issues worth focusing on corresponding to the above insertion mechanisms, such as the capacity division problem of multiple ions, the hybrid insertion mechanism of dual ions, and the high electrolyte concentration and H^+^-free action of single ions. The outermost layer is a summary of the main ideas, which are rechargeable zinc-ion batteries and ionic insertion mechanisms. Ions play a crucial role in aqueous rechargeable zinc batteries, affecting performance and cycle life. This review first summarizes the mechanisms of different ions in these batteries. Then, the mechanisms are systematically classified and comparatively analyzed. Finally, the effective utilization of different ions is discussed to guide the development of water-based rechargeable zinc batteries in the future.

## 2. Ion Insertion Mechanism of Rechargeable Zinc Batteries

**Figure 2:**
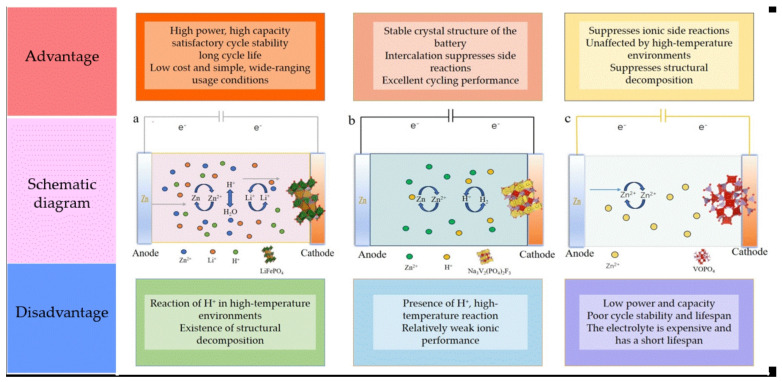
Schematic diagram of different batteries and their advantages and disadvantages. (**a**) Schematic diagram of Zn-LiFePO_4_ aqueous rechargeable battery; (**b**) schematic diagram of Zn-Na_3_V_2_(PO_4_)_2_F_3_ aqueous rechargeable battery; and (**c**) schematic diagram of Zn-VOPO_4_ rechargeable battery in the electrolyte 21 M LiTFSI/1 M Zn (Tr)_2_ solution.

As shown in Figure 2, the schematic diagrams of batteries with different cationic interactions are presented, along with their advantages and disadvantages. Among them, as shown in the middle row, is the schematic diagram of multi-ion insertion, dual-ion insertion, and single-ion insertion batteries; the row above is the corresponding advantages of these three insertion mechanisms; and the row below is their corresponding disadvantages. The comparison of batteries with different insertion mechanisms provides theoretical support for our discussion. In the case of multiple ions acting together, H^+^ is present. In contrast, with single-ion action, the active element in the cell is free of H^+^, with Zn^2^^+^ as its main component. Due to the roles of different ions, the battery exhibits various electrochemical properties. Common cathode materials for zinc-ion batteries and their electrochemical properties are shown in Table 2. There is a significant difference in the performance of different cathode materials under the same electrolyte, and the same cathode material can exhibit varying properties under different electrolytes. Therefore, when exploring battery performance, it is challenging to initially determine the main conditions affecting it due to the variety of factors at play. To facilitate discussion, ion interactions in rechargeable zinc batteries can be categorized into three types: co-action of multiple cations, co-action of dual cations, and the action of a single cation.

### 2.1. Multi-Ion Insertion Mechanism of Rechargeable Zinc Batteries

During the charge and discharge processes of rechargeable zinc batteries, multiple ions interact simultaneously. The behavior and properties of ions inside batteries are influenced by multiple factors [31]. Therefore, it is necessary to consider the interactions and effects of various ions comprehensively when discussing the battery mechanism. The electrochemical charge storage mechanism in aqueous zinc-ion batteries is generally the reversible insertion of Zn^2+^ into the host material [32]. This is also the primary mechanism for most polyanion compounds. As shown in Figure 2a, in the rechargeable battery composed of LiFePO_4_ and Zn, during discharge, Li^+^ ions in the mixed electrolyte generate LiFePO_4_ and insert into the heterostructure (FePO_4_). Simultaneously, zinc metal loses electrons, forming Zn^2+^ ions that migrate back to the electrolyte.

The primary working mechanism of Zn-LiFePO_4_ can be represented by the following equations:

Cathode, LiFePO_4_ ↔ e^−^ + Li^+^ + FePO_4_; (1)

Anode, Zn^2+^ + 2e^−^ ↔ Zn; (2)

Overall, 2LiFePO_4_ + Zn^2+^ ↔ 2Li^+^ + 2FePO_4_ + Zn. (3)

Research has found that in aqueous electrolytes with polyanion compounds, an H^+^ (or Na^+^) insertion often occurs alongside a Zn^2+^ insertion [33]. However, many studies on aqueous zinc-ion batteries do not report on H^+^. Despite this, H^+^ is commonly present in aqueous batteries. For instance, in the Zn-LiFePO_4_ battery shown in Figure 2a, an analysis of its electrode potential reveals the presence of H^+^. Therefore, during the charge and discharge processes, Zn^2+^, Li^+^, and H^+^ ions interact simultaneously. Although Zn^2+^ usually predominates over H^+^, there are cases where H^+^ plays a similar or even dominant role compared to Zn^2+^ and should not be ignored [34]. For example, Wan et al. [28], by analyzing the differential capacity curve (dQ/dV) of a layered VOPO_4_·xH_2_O cathode in different electrolytes, found that H^+^ intercalation dominates in an electrolyte composed of 5 m ZnCl_2_/0.8 m H_3_PO_4_. Therefore, when discussing the multi-ion insertion mechanism, it is necessary to clarify which ion plays the primary role among the multiple ions. Sometimes, it is also necessary to discuss the situation in which different ions act under different conditions in the same battery. Park et al. [35] studied the differences in the electrochemical behavior of Na_3_V_2_(PO_4_)_2_F_3_ as a cathode material in non-aqueous and aqueous Zn-ion batteries (ZIBs). Their in situ analysis revealed that the observed differences in electrochemical behavior were due to different storage mechanisms. In non-aqueous ZIBs, Zn^2+^ and Na^+^ were initially identified as guest ions, but gradually, only Zn^2+^ was present. In contrast, in aqueous ZIBs, H^+^ was found to be the dominant guest ion instead of Zn^2+^. Therefore, the multi-ion insertion mechanism of the battery should be discussed under various conditions to inspire further exploration of the battery’s insertion mechanisms.

### 2.2. Dual-Ion Insertion Mechanism of Rechargeable Zinc Batteries

In addition to the multi-ion insertion mechanism, aqueous zinc-ion batteries also exhibit a dual-ion insertion mechanism, in which Zn^2+^ and another ion act together. This mechanism is simpler than the multi-ion insertion mechanism, as it involves fewer ionic embedding and de-embedding processes. Consequently, it has gained increasing attention. In aqueous zinc-ion batteries, H^+^ is inevitable due to the electrolyte’s characteristics, so it must be considered in our discussion. Research has shown that in the electrochemical reaction of open-tunnel or layered cathode materials, there is not only the classical Zn^2+^ insertion/extraction mechanism but also a H^+^/Zn^2+^ co-insertion/extraction mechanism [36]. The aqueous Zn-Na_3_V_2_(PO_4_)_2_F_3_ rechargeable battery in Figure 2b is a typical battery with Zn^2+^/H^+^ co-insertion. Li et al. [26] synthesized the carbon nanotube-coated Na_3_V_2_(PO_4_)_2_F_3_ aqueous zinc-ion battery (AZIB) anode material with a continuous and interconnected ion transport channel structure. X-ray diffraction (XRD) and X-ray photoelectron spectroscopy (XPS) revealed the Zn^2+^/H^+^ co-intercalation mechanism. The specific mechanism of the reaction was found to be as follows:

During the first charging process: electrochemical reaction,

Na_3_(VO)_2_(PO_4_)_2_F ↔ Na_x_(VO)_2_(PO_4_)_2_F + (3 − x)Na^+^ + (3 − x)e^−^ (4)

with the chemical reaction

Na_3_(VO)_2_(PO_4_)_2_F ↔ VOPO_4_ + amorphous byproducts, (5)

and for electrochemical reactions during subsequent charging and discharging processes,

Na_x_(VO)_2_(PO_4_)_2_F + yZn^2+^ + z H^+^ + (2y + z)e^−^ ↔ Na_x_Zn_y_H_z_(VO)_2_(PO_4_)_2_F (6)

VOPO_4_ + aZn^2+^ + bH^+^ + (2a + b)e^−^ ↔ Zn_a_H_b_VOPO_4_, (7)

The reactions indicate the presence of a Zn^2+^/H^+^ co-insertion layer in the cell, with the electrochemical mechanism involving Zn^2+^ insertion followed by H^+^ co-insertion. Optimizing the material structure and morphology and adjusting the electrolyte composition provides new insights for related research. The dual-ion insertion mechanism simplifies the complex interactions of multiple ions, enhancing ion embedding and de-embedding, and improving battery stability. Consequently, the dual-ion co-insertion approach has been progressively refined and adopted in various batteries. For instance, researchers have observed significant Zn^2+^/H^+^ co-insertion in oxide cathodes cycled in aqueous media, whereas Zn^2+^ ions alone often prefer non-oxide materials. This difference may be due to the interface nature, which is typically hydroxyl-terminated and hydrated in most oxides and phosphates in aqueous media [37]. Wang et al. reported a novel vanadium-based oxide cathode based on MgV_2_O_6_⋅1.7H_2_O nanoribbons. Analysis by X-ray photoelectron spectroscopy (XPS), X-ray diffraction (XRD), and inductively coupled plasma optical emission spectroscopy (ICP-OES) revealed an irreversible Mg^2+^-Zn^2+^ ion-exchange reaction during the initial discharge, followed by an unusual H^+^/Zn^2+^ intercalation reaction. In addition, Tao et al. [38] verified Zn^2+^/H^+^ co-insertion/extraction in hydrated zinc vanadium oxide/carbon cloth (ZnVOH/CC) electrodes by off-site transmission electron microscopy (TEM), off-site XPS, and other characterization methods. These findings demonstrate the practical application of the H^+^/Zn^2+^ bi-ionic insertion mechanism, paving the way for further exploration of ionic intercalation in batteries.

### 2.3. Mechanism of Single-Ion Insertion in Rechargeable Zinc Batteries

H^+^/Zn^2+^ co-embedding occurs at the cathode of zinc-ion batteries, but methods to inhibit harmful H^+^ embedding are limited [34]. Despite an increased focus on aqueous zinc-ion batteries, several issues have emerged. H^+^ ion embedding can balance the insertion and removal processes of Zn^2+^ ions, reduce zinc dendrite formation and electrode polarization, and enhance cycling stability and battery lifetime. However, the H^+^ embedding mechanism poses challenges. First, H^+^ ion embedding can alter the electrode material’s structure, causing volume expansion and contraction, which may lead to material breakage and deactivation. Second, side reactions from water decomposition can generate gases, increasing internal gas pressure. Additionally, H^+^ poses safety concerns, especially in high-temperature scenarios, and proton insertion can form layered double hydroxide salts (LDHs), such as Zn_4_SO_4_(OH)_6_·5H_2_O, on the surface of metal oxides [32]. These LDHs form an insulating layer that detaches from the electrode over time, leading to active material loss. To address these issues, there has been a growing emphasis on single-ion insertion mechanisms that exclude H^+^ embedding. To inhibit H^+^ action, modifications to the electrolyte components are often considered, which is why highly concentrated electrolytes have gained attention [39].

Taking Figure 2c as an example, the Zn/VOPO_4_ aqueous cell uses a 21 M LiTFSI/1 M Zn(Tr)_2_ solution as the electrolyte, effectively inhibiting H^+^ activity within the cell. The energy storage mechanism can be summarized as follows: in the low voltage region (0.8–1.8 V), Zn^2+^ ions are inserted into and extracted from VOPO_4_. The redox process of lattice oxygen atoms in VOPO_4_ is not required for the Zn^2+^ ion insertion/extraction but is instead primarily involved in the insertion/extraction of other ions between 1.8 and 2.1 V [27]. Its reaction mechanism is summarized as follows:

Cathode, Zn_x_VOPO_4−_xZn^2+^ − (2x + y)e^−^ ↔ VO^y+^PO_4_; (8)

Anode, (x + y/2)Zn^2+^ + (x + y/2)e^−^ ↔ (x + y/2)Zn; (9)

Overall, Zn_x_VOPO_4_ + y/2Zn^2+^ ↔ VO^y+^PO_4_ + (x + y/2)Zn^2+^. (10)

By altering the battery’s electrolyte, not only was the role of H^+^ intercalation modified, but the reversibility of the crystal structure transformation in VOPO_4_ during charge/discharge cycles was also enhanced through redox reactions. This improvement resulted in excellent capacity retention and a long-term cycle life. This encouraged us to inhibit the role of H^+^ by controlling a series of factors, such as the type of electrolyte and even pH, to ultimately achieve a benign application for the battery mechanism.

## 3. Ionic Properties of Rechargeable Zinc Batteries

In the previous sections, the effects of various ion embedding and de-embedding on rechargeable zinc batteries were examined. The primary focus of subsequent research should be on how these mechanisms impact battery performance. Our analysis reveals that different ion interactions significantly influence the performance of zinc-ion batteries. To facilitate this analysis, this paper categorizes the ionic effects into three types based on previous work: multi-ionic interaction performance, dual-ionic interaction performance, and single-ionic interaction performance.

### 3.1. Multi-Ion Interaction Properties

Due to the widespread use of aqueous electrolytes, H^+^ is unavoidable in batteries; consequently, multi-ion insertion is commonly observed in aqueous zinc-ion batteries [40]. Moreover, due to multi-ion insertion, the performance of batteries is influenced by various factors, complicating the situation within the batteries. To distinguish the factors affecting battery performance, it is essential to compare different batteries to obtain results that meet our needs and identify the main factors influencing battery performance.

**Figure 3:**
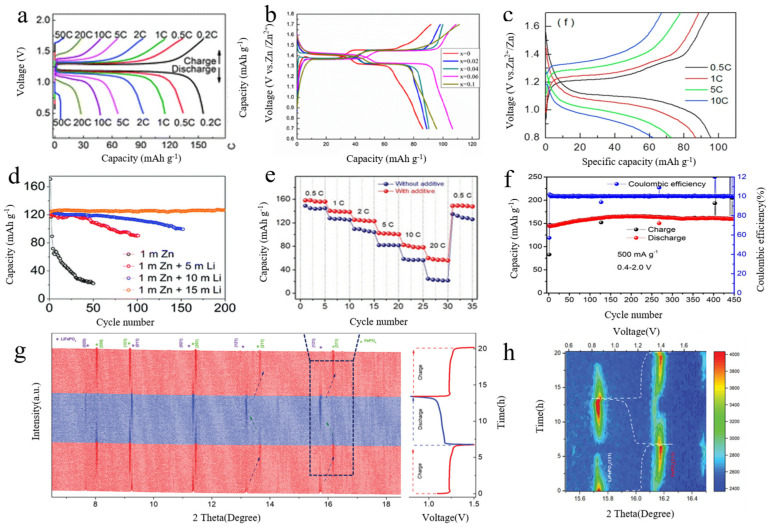
(**a**) Charge-discharge curves of Zn-LiFePO_4_ [3]; Copyright© 2013 Copyright Clearance Center, Inc. All rights reserved, United Kingdom of Great Britain and Northern Ireland (**b**) Initial charge–discharge curves of Li_3_V_2−x_Mn_x_(PO_4_)_3_ (x = 0.00, 0.02, 0.04, 0.06, 0.1) [41]; Copyright© 2023 Advanced Energy Materials, published by Wiley-VCH GmbH, American (**c**) Charge–discharge curves of Zn//0.5 mol L^−1^ + Zn(CH_3_COO)_2_//Na_3_V_2_(PO_4_)_3_ cell charge/discharge curves [42]; Copyright © 2022, under exclusive license to Springer-Verlag GmbH Germany, part of Springer Nature, Germany (**d**) Cycling stability of Li_3_V_2_(PO_4_)_3_ cathode with different electrolytes; Copyright© 2016 Elsevier Ltd. All rights reserved. the Netherlands (**e**) Rate capability of LiFePO_4_ tested in the range of 0.5 to 20 C [43]; Copyright© 2024 Copyright Clearance Center, Inc. All rights reserved, United Kingdom of Great Britain and Northern Ireland (**f**) Cycling performance at 500 mA·g^−1^ in the potential range of Na_3_V_2_(PO_4_)_3_@C of 0.4~2.0 V [17]. Copyright© 2021, American Chemical Society, American (**g**) The operando synchrotron XRD patterns of the hybrid Zn-LiFePO_4_ (left) and the corresponding charge–discharge curve (right) [43]. (**h**) Contour plots of the operando synchrotron XRD data, 15.5–16.5°, in which the (131) peak of LiFePO_4_ is converted to the (311) peak of FePO_4_ during the initial charge process [43]. Copyright© 2024 Copyright Clearance Center, Inc. All rights reserved, United Kingdom of Great Britain and Northern Ireland.

Figure 3a shows the charge–discharge curves of the Zn//CH_3_COOLi+ Zn(CH_3_COO)_2_//LiFePO_4_ hybrid battery at different current rates. The capacity at different rates exhibits a regular gradient change, indicating that the Zn-LiFePO_4_ system demonstrates stability. The perfect recovery of capacity and high efficiency close to 100% indicate that the system has excellent rate performance and a long cycle life, with a continuous current above 20 °C suggesting substantial power output. During charging, Li^+^ is de-inserted from the FePO_4_ matrix, Zn^2+^ is deposited from the electrolyte, and electrons are gained from the current collector. Conversely, during discharge, the opposite process occurs. The associated processes are illustrated in Equations (1)−(3). The system can provide an output voltage of about 1.2 V, with a high expected capacity, satisfactory rate performance, and long cycle life at temperatures above 20 °C. The aqueous zinc battery exhibits a range of excellent properties, and the insertion and de-embedding of various ions enable the battery to achieve substantial power output.

Adding high-entropy elements to the battery can also significantly improve its electrochemical performance. High-entropy materials (HEMs), which are single-phase crystal structures composed of many different elements, open up a huge space of chemical parameters with an almost unlimited number of element combinations. This versatility enables compounds to meet specific needs and be tailored to desired properties and applications, improving the quality and functionality of the material in a sustainable way. It has broad application prospects because its properties can be adjusted by selecting specific elements and changing the stoichiometry. Appropriate doping of metal elements in the negative electrode can alter the inter-crystalline spacing of the cathode material, alleviate crystal deformation, and enhance conductivity and ion diffusion rates, thereby affecting battery performance. As shown in Figure 3b, metal Mn was doped into the negative electrode Li_3_V_2_(PO_4_)_3_ of a zinc battery, and the initial charge/discharge curves were analyzed. The capacities of the battery with Zn//Li_3_V_2−x_Mn_x_(PO_4_)_3_ (x = 0.00, 0.02, 0.04, 0.06, and 0.1) were found to be 86.5 mAh·g^−1^ (x = 0), 89 mAh·g^−1^ (x = 0.02), 90 mAh·g^−1^ (x = 0.04), 106 mAh·g^−1^ (x = 0.06), and 95.5 mAh·g^−1^ (x = 0.1) The cell capacity gradually increased with an increase of Mn content, a result attributed to the improvement of the interlayer structure and equilibrium charge of the material by proper Mn doping.

Figure 3c shows the current density of the charge/discharge curves for the Zn-Na_3_V_2_(PO_4_)_3_ cell under different operating conditions. The specific discharge capacities are 97, 89, 79, and 58 mAh·g^−1^ at 0.5, 1, 5, and 10 C, respectively. The figure indicates that the electrochemical performance of the cell is excellent, allowing it to be charged and discharged at high rates up to 10 C. The specific discharge capacity of the Zn-Na_3_V_2_(PO_4_)_3_ cell is illustrated in Figure 3c. According to Equations (4)–(7), the battery undergoes charging and discharging involving not only Zn ions but also the embedding and de-embedding of Na ions, along with the valence change of V elements. Their combined action results in good battery performance, suggesting that the interaction between the two cations is significant, prompting further investigation into the mechanism of the aqueous zinc-ion battery.

Cyclic stability is a key performance indicator for electrochemical energy storage and conversion systems. It measures an electrochemical device’s ability to maintain stable performance, including capacity, efficiency, and internal resistance, during repeated charging, discharging, or usage. Good cyclic stability is crucial for the long-term reliability of electrochemical systems. It enhances service life, ensures safety and reliability, increases energy density, and reduces maintenance costs. Figure 3d–f illustrate the cyclic stability of various batteries.

The electrolyte plays a crucial role in determining cycling stability [44]. Figure 3d illustrates the cycling performance of the Li_3_V_2_(PO_4_)_3_ cathode at 200 mA·g^−^¹ in various aqueous solutions. The study compared 1 M Zn, 1 M Zn + 5 M Li, 1 M Zn + 10 M Li, and 1 M Zn + 15 M Li, evaluating how different Li concentrations affected cycling stability. The results showed that cycling stability improved with increasing Li concentrations. Notably, the 1 M Zn + 15 M Li system exhibited the highest cycling stability, achieving a reversible capacity of 126.7 mAh·g^−^¹ after 200 cycles without significant degradation. The Coulombic efficiency (CE) reached 99.8%. This improvement is attributed to enhanced Li^+^ insertion and de-embedding with higher Li concentrations, which likely contributes to the increased cycling stability. The Zn/Li_3_V_2_(PO_4_)_3_ cell showed reversible Li^+^ insertion into the polyanion cathode during cycling, while Zn^2+^ plating and stripping occurred at the anode. These findings suggest that optimizing electrodes and electrolytes could further improve the performance of rechargeable Zn-based batteries.

The addition of additives can also impact the cycling stability of the battery [45]. Figure 3e illustrates the performance of Zn/LiFePO_4_ batteries with and without additives. Research studies identified sodium dodecylbenzene sulfonate (SDBS) as an additive that enhances the electrochemical behavior of Zn/LiFePO_4_ hybrid batteries. Applying this additive to the Zn/LiFePO_4_ cell improved its performance. When evaluating cycling stability, it was found that at a high current rate of 20 C, the capacity of the Zn/LiFePO_4_ cell without the additive was only 22.5 mAh·g^−^¹, with a capacity retention of about 15.3% compared to 0.5 C. In contrast, the cell with the electrolyte additive maintained a capacity of 57.8 mAh·g^−^¹ at the same current rate. Further analysis revealed that this additive not only suppressed Zn dendrite growth by controlling the Zn plating pattern but also accelerated lithium-ion diffusion at the LiFePO_4_ cathode/electrolyte interface. With the additive, Zn^2+^ ions were smoothly deposited on the Zn metal surface, preventing lamellar Zn dendrite growth. Additionally, the additive improved the wettability of the LiFePO_4_ electrode, increasing the lithium ion diffusion coefficient from 1.78 × 10^−^¹¹ to 8.22 × 10^−^¹¹ cm^2^ s^−^¹, thus enhancing the cell’s performance at high rates. This suggests that the main mechanism of the additive is to facilitate ion diffusion at the interface through its surface-active properties. Both Zn^2+^ and Li^+^ ions play crucial roles at the anode and cathode, respectively. Therefore, in optimizing battery performance, it is important to consider both the electrolyte and its additives.

In addition to the factors mentioned above, structural decomposition reactions occurring in the electrolyte during the cycling process also influence ion diffusion within the cell, which in turn affects the electrochemical performance [46]. Unlike the Zn-Na_3_V_2_(PO_4_)_3_ cell structure and reaction mechanism discussed earlier, materials in a 1 M Zn(CF_3_SO_3_)_2_ aqueous solution were found to undergo unexpected structural decomposition reactions during prolonged cycling. Specifically, Na_3_V_2_(PO_4_)_3_ decomposes into vanadium oxides such as Zn_3_V_2_O_8_, V_2_O_5_, and VO_2_. This decomposition leads to significant changes in the charge/discharge plateau, reversible capacity, kinetics, and structural stability. Notably, cycling stability, which is a primary concern, is also greatly impacted. As shown in Figure 3f, when Na_3_V_2_(PO_4_)_3_@C is cycled further at 500 mA·g^−1^ within the range of 0.4 to 2.0 V, the reversible capacity initially increases to 145 mAh·g^−1^ and stabilizes at 160 mAh·g^−1^ after 450 cycles. Subsequent studies revealed that the structure and morphology of zinc remain stable post-cycling, indicating that Na_3_V_2_(PO_4_)_3_@C undergoes phase changes due to the cumulative effects of repeated cycling. Thus, structural decomposition reactions in the electrolyte can significantly affect the cycling stability of the battery.

The charge relationship inside the battery also has a profound impact on the mechanism of the battery, which in turn affects the performance of the battery. The phase evolution of LFP during charge and discharge of mixed Zn/LiFePO_4_ cells was studied by XRD of the atom synchrotron. From the charge–discharge curve (Figure 3g), only one discharge platform located at ≈1.1 V can be observed, indicating a single-phase transition. Acyclic LiFePO_4_ has distinct peaks at 7.7°, 9.2°, 11.4°, and 13.2° and can be found in the (020), (011), (021), and (121) planes, respectively. During charging, these peaks gradually disappeared, while new reflections of ≈8.0°, 9.2°, 11.5°, and 13.8° appeared, which can be used for 200, 101, 201, and 211 reflections of the FePO_4_ phase, indicating a complete transformation of the LFP phase into a heterogeneous phase. After discharge, all LiFePO_4_ peaks reappeared completely, while the crystalline phase of FePO_4_ disappeared, indicating good phase reversibility. A more pronounced change can be observed in the contour plot (Figure 3h), which shows that only Li ions and not Zn^2+^ ions are inserted into FePO_4_ during discharge, although the size of Li ions is slightly larger than that of Zn^2+^ ions. The main reason can be attributed to the properties of Zn^2+^ and Li ions: compared to Li ions, Zn^2+^ ions have a higher charge density (double charge divided by a small radius of 0.74 A), resulting in reduced diffusivity within the body of ordinary polar crystals due to strong coulomb interactions and inhibition problems related to electrolyte chemistry, affecting the implantation/stripping solvation/desolvation energy balance.

### 3.2. Dual Ionization Properties

Dual-ion insertion is also widely used in batteries, and its effects on performance merit exploration. Unlike multiple-ion insertion, the impact of H^+^ in dual-ion insertion cannot be ignored, leading to different effects on battery performance [47]. Consequently, research in this area has different focuses compared to multiple-ion insertion. The primary challenge for this mechanism is addressing the effects of H^+^. Many researchers have proposed solutions to this issue.

**Figure 4:**
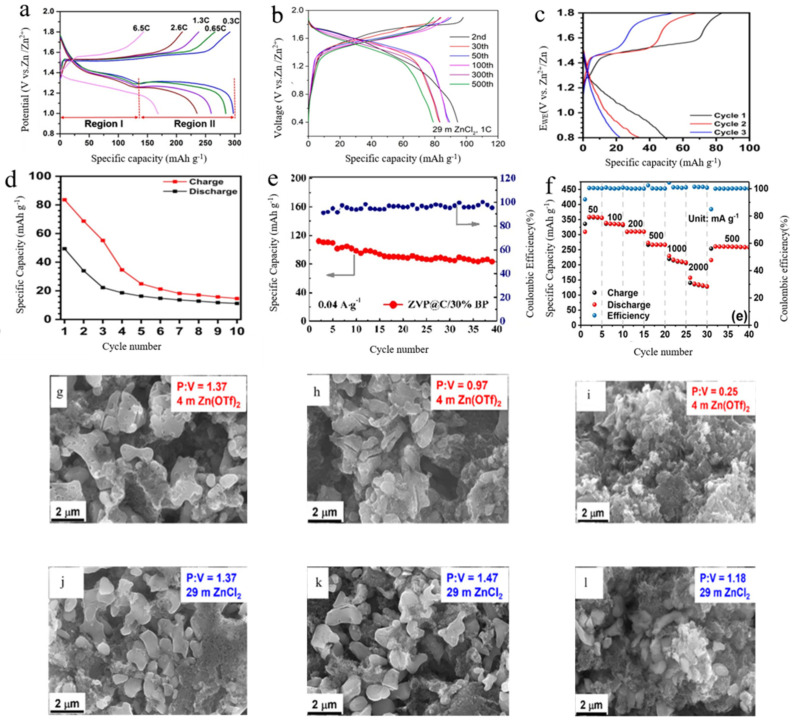
(**a**) Charge/discharge curves at different rates in the first cycle; Copyright© 2017, American Chemical Society, American (**b**) Charge/discharge curves of Li_3_V_2_(PO_4_)_3_ during different cycles; Copyright© 2022, American Chemical Society, American (**c**) Charge/discharge curves of Zn/Na_3_V_2_(PO_4_)_2_F_3_ at 0.3 C; Copyright© 2020, American Chemical Society, American (**d**) Cycling performance of Na_3_V_2_(PO_4_)_2_F_3_/C in non-aqueous zinc-ion batteries at 0.3 C; Copyright© 2020, American Chemical Society. (**e**) Cycling stability of Zn_3_V_4_(PO_4_)_6_ starting from the second cycle at 0.04 Ag^−1^ @C/30%BP cycle stability [48]; Copyright© 2022, American Chemical Society, American (**f**) Specific capacity and coulombic efficiency obtained at different specific currents [49]. Copyright © 2020 American Chemical Society, American (**g**–**l**) Corresponding SEM images and P:V ratios collected by EDX at the 2nd (**g**,**j**), 5th (**h**,**k**), and 20th (**i**,**l**) cycle; Copyright© 2022, American Chemical Society, American.

Compared to multi-ion action, the properties of dual-ion action are different due to the ions. As shown in Figure 4a, the Zn/MnO_2_ charge–discharge curve shows @ carbon fiber paper (CFP) cells at different rates in the first cycle. When the charge and discharge rate is increased from 0.3 C to 6.5 C, about 60% of the capacity can be maintained at 0.3 C, showing excellent magnifying capacity. Interestingly, with an increase of the charge and discharge rate, the voltage and capacity of the first voltage platform (region I) decreased very little, while the voltage and capacity of the second voltage platform (region II) decreased significantly, indicating that the reaction kinetics of the first (high) platform were much faster than those of the second (low) platform. At a high rate of 6.5 °C, the reaction of the second platform accounts for less than 20% of the total capacity. This significant difference in kinetics between the two reaction regions can also be observed by cyclic voltammetry (CV) scanning at different rates.

Li et al. [34] demonstrated that engineering the solvated structure of aqueous Zn electrolytes (AZEs) is an effective method to inhibit H^+^ intercalation and promote dominant Zn^2+^ intercalation. To validate this concept, they selected Li_3_V_2_(PO_4_)_3_ as the cathode and compared its electrochemical performance using two previously reported electrolytes: 4 M Zn (OTf)_2_ and 29 M ZnCl_2_. They also evaluated a newly designed hybrid electrolyte comprising poly (ethylene glycol) 400 (Polyethylene glycol (PEG) 400) and water as co-solvents, with Zn(OTf)_2_ as the salt. As shown in Figure 4b, ZnCl_2_ did not exhibit a low-voltage plateau but only a smaller activation-like process. In contrast to the maximum discharge capacity of 200 mAh·g^−^¹ observed with 4 M Zn(OTf)_2_, ZnCl_2_ achieved a discharge capacity of approximately mAh·g^−^¹ at the 60th cycle after the initial activation process. Figure 4g–l shows that the morphology and P:V ratio of Li_3_V_2_(PO_4_)_3_ electrodes only change slightly in ZnCl_2_ WiSE, in which clear and distinguishable crystalline particles are preserved upon cycling. The presence of H^+^ in the cell, due to the electrolyte’s role in charge transfer and storage, necessitates the suppression of H^+^ to ensure safety and prevent potential fire hazards.

To address the issue of H^+^ intercalation, the use of non-aqueous electrolytes can be considered. As shown in Figure 4c, the Na_3_V_2_(PO_4_)_2_F_3_/C cathode exhibits poor reversibility in non-aqueous Zn-ion batteries. Constant current cycling at a lower rate of 0.3 C reveals that the initial discharge capacity of the cell is only 49 mAh·g^−^¹. Furthermore, Figure 4d shows that this capacity decreases sharply during the first three cycles and gradually stabilizes at 11 mAh·g^−^¹ by the end of 10 cycles. The non-aqueous Zn-ion battery with Na_3_V_2_(PO_4_)_2_F_3_/C exhibits a significantly shorter cycle life (10 cycles) compared to the aqueous cell (30 cycles). These results indicate notable differences in electrochemical behavior between aqueous and non-aqueous systems. While non-aqueous systems have some drawbacks in terms of recyclability, aqueous batteries offer unique advantages.

In contrast, Zhao et al. [48] synthesized a novel rocking-chair AZIB cathode material, Zn_3_V_4_(PO_4_)_6_@C (ZVP@C), and evaluated its electronic conductivity with a composite carbon coating. According to Figure 4e, ZVP@C/30% BP provides good stability even at current densities as low as 40 mA·g^−1^. This stability is attributed to the two-electron reaction of vanadium and the co-intercalation of Zn^2^^+^/H^+^. The capacity retention of Zn^2^^+^/H^+^ reached 80% after 400 cycles at 1 A·g^−1^, which was due to the stabilization of the crystal structure and the co-intercalation reaction of Zn^2^^+^/H^+^.

The electrochemical performance of high-performance δ-calcium vanadium oxide bronze/reduced graphene oxide (CVO/rGO) as an AZMB cathode material was evaluated using Zn foil as the negative electrode, 3 M Zn(OTf)_2_ as the electrolyte, and an aqueous solution in a button cell [49]. The specific capacities at various current densities measured by GCPL are shown in Figure 4f. Specific capacities of 267 and 215 mAh·g^−1^ were achieved at current densities of 500 and 1000 mA·g^−1^, respectively. This figure demonstrates the good cyclability of the cell. The CVO/rGO composite exhibits satisfactory performance as a cathode material and is suitable for studying the insertion mechanism.

Summarizing the performance of bi-ionic insertion, it can be observed that co-intercalation is an effective method to mitigate the impact of H^+^ on battery performance. Although a simpler approach is to inhibit or even eliminate H^+^ by changing the electrolyte, this will subsequently alter the battery’s performance.

### 3.3. Single-Ion Performance

In general, the main difference between single-ion action and multi-ion action is that to avoid the presence of H^+^, a non-aqueous electrolyte is preferred, often a high-concentration electrolyte using the “water-in-salt” concept. This inhibits H^+^ activity, reduces multi-ionic interactions, and establishes a foundation for further optimization of the ion insertion mechanism [50]. Therefore, single-ion interactions also possess distinct characteristics affecting battery performance.

**Figure 5:**
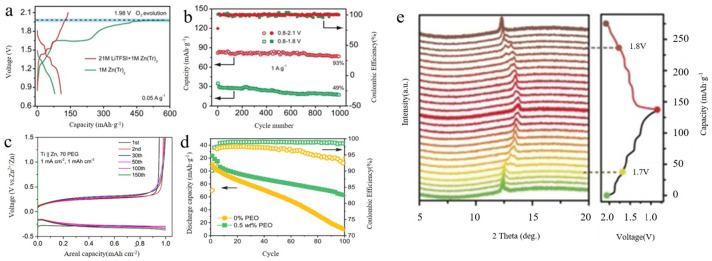
(**a**) First charge/discharge curves of Zn/VOPO_4_-based batteries with different electrolytes; Copyright© 2019 Wiley-VCH Verlag GmbH & Co. KGaA, Weinheim, Germany. (**b**) Cycling performance of batteries employing the electrolyte in different voltage windows of 21 M LiTFSI/1 m Zn(Tr)_2_; Copyright© 2019 Wiley-VCH Verlag GmbH & Co. KGaA, Weinheim, Germany. (**c**) Charge/discharge curves of Zn asymmetric cells with Ti||1 mA^−2^ at 70 PEG [34]; Copyright© 2022, American Chemical Society, Washington, WA, USA. (**d**) Cycling stability and Coulombic efficiency of a full cell with electrolyte with or without PEO additive, 1 M ZnSO_4_ in 0.5 C [51]; Copyright© 2020 Wiley-VCH GmbH. (**e**) In situ XRD patterns of VOPO4/SWCNT electrodes; Copyright© 2019 Wiley-VCH Verlag GmbH & Co. KGaA, Weinheim, Germany.

In the 21 M LiTFSI/1 M Zn(Tr)_2_ (aqueous salt) electrolyte, the O_2_ precipitation reaction occurs even when charged to 2.10 V (Figure 5a). Additionally, the high concentration of the 21 M LiTFSI/1 M Zn(Tr)_2_ electrolyte inhibits the dissolution of VOPO_4_ and the corrosion of the Zn anode by reducing water activity. While the high electrolyte concentration can limit excessive ion reactions, it also affects cell performance.

Under 5 A·g^−1^ conditions, the capacity retention after 1000 cycles was as high as 93% when the voltage window was 0.8~2.1 V (Figure 5b). In contrast, when the voltage window was 0.8~1.8 V, the corresponding capacity of the Zn/VOPO_4_ cell was only 20 mAh·g^−1^, and the capacity retention after 1000 cycles was only 49%. This indicates that the high concentration of electrolyte imposes certain requirements on the operating conditions of the battery.

By evaluating the Ti||Zn asymmetric cell, a Coulombic efficiency of about 99.7% can be achieved by 70 PEG at a current density of 1 mA cm^−2^, as shown in Figure 5c. In contrast, 0 PEG cells form extensive dendrites under the same operating conditions and fail after three cycles. The Coulombic efficiency of the cell can be improved to some extent by additives.

In situ XRD was performed during a typical charge/discharge cycle to further understand the crystal structure evolution of VOPO_4_ (Figure 5e). During the discharge process from 1.7 to 0.8 V (vanadium reduction), three lattice distortions occurred, as suggested by the successive appearance of three new peaks, in accordance with the three discharge plateaus in the low-voltage region. However, the crystal structure of VOPO_4_ at the charged state of 1.8 V did not recover fully to the corresponding discharged state of 1.7 V, thus demonstrating that the crystal structure evolution was not completely reversible in the voltage window of 0.8–1.8 V. Therefore, the Zn/VOPO_4_ batteries display unsatisfactory cycling performance from 0.8 to 1.8 V. Impressively, when the batteries were charged to 2.1 V, the crystal structure of VOPO_4_ recovered to the initial state. Thus, when the voltage window was 0.8–2.1, the Zn/VOPO_4_ batteries displayed excellent cycling performance.

With the addition of 0.5 wt% Polyethylene oxide (PEO), the battery can be safely charged to a higher voltage of 2.05 V, while Zn achieved a higher capacity of 125 mAh·g^−^¹. (Note: The higher oxidative settling voltage of the PEO-containing electrolyte is partly due to the increase in polarization on the Zn anode (≈<50 mV). The use of Zn/LiMn_2_O_4_ cells with PEO additives shows significantly improved capacity retention (Figure 5d). Additionally, the Coulombic efficiency (CE) degraded rapidly to 92% in the PEO-free electrolyte over 100 cycles, whereas it remained as high as 99% in the Zn/LiMn_2_O_4_ cells with a 0.5 wt% PEO electrolyte additive. This improvement can be attributed to the stabilized Zn anode and suppression of gas generation in the PEO-containing electrolyte. Mono-ion batteries enhance safety by addressing the issue of the H^+^ presence through the use of high-concentration electrolytes.

From the above discussion, it can be seen that single-ion batteries differ from other types of batteries in terms of performance. The electrochemical performance is inferior to other batteries, but it has safety and stability. It is clear that numerous factors influence the electrochemical performance of the battery, ranging from characteristics to mechanisms. Therefore, in further discussions, it is essential to consider these various factors to achieve a clearer understanding of the battery’s energy storage mechanism.

## 4. Opportunities and Challenges

During the exploration of different batteries, it was found that rechargeable batteries utilizing multivalent ions can theoretically offer higher storage capacities due to multiple electron transfers. Aqueous zinc-ion batteries (ZIBs) based on Zn^2+^ intercalation chemistry have gained significant attention due to their zinc anodes, which provide high capacity, high abundance, low cost, and low redox potentials [52]. However, due to the nature of the electrolyte in aqueous zinc-ion batteries, H^+^ is inevitably present and affects the storage and transfer of electrons [53]. According to the comparison of the charge and discharge curve and the cyclic stability curve above, we can try to summarize the influence of H^+^ on the electrochemical performance of the battery and the methods for avoiding or reducing such influence. On the one hand, the presence of H^+^ can enhance ion conductivity, balance the insertion and removal process of Zn^2+^ ions, reduce the formation of zinc dendrites and electrode polarization, and improve cycle stability and battery life. Therefore, in the past development process of batteries, aqueous electrolytes have been favored by many researchers. On the other hand, although people are increasingly concerned about water-based zinc-ion batteries, there are also some problems. First, H^+^ ion embedding changes the structure of the electrode material, causing volume expansion and contraction, which may lead to fracture and deactivation of the material. Second, the side reaction of water decomposition will produce gas, increasing the internal gas pressure. Finally, H^+^ may have certain safety risks under high temperature conditions, which will cause great risks in the industrial production process, so it should be avoided as much as possible. Therefore, methods to inhibit the action of H^+^ have garnered interest, leading to the development of batteries with single-ion insertion [54]. Summarizing the conclusions of the above literature, we can find strategies to solve or avoid the above problems. H^+^ insertion can be inhibited by engineering the solvation structure of aqueous zinc electrolytes (AZEs). Co-insertion is an effective method to alleviate the effect of hydrogen ions on battery performance. In order to avoid the presence of H^+^, a non-aqueous electrolyte is preferred, usually a high-concentration electrolyte that uses the concept of “water in salt”. This inhibits H^+^ activity and reduces multi-ion interactions, laying a foundation for further optimization of ion insertion mechanisms.

In fact, before the widespread use of non-aqueous solvents began in the 1950s, water was the most readily available and almost the only solvent used in all areas of pure and applied chemistry [55]. However, as batteries continue to evolve, their application scenarios are expanding, leading to the continuous development of various battery types. Consequently, non-aqueous zinc batteries are advancing. To address issues such as dendrite formation and simultaneous water splitting during the charge–discharge process, and to improve Coulombic efficiency and cycling stability, water-in-salt electrolytes have been developed and are increasingly used in batteries [56]. These electrolytes not only solve the aforementioned problems but also inhibit the production of H^+^. As a result, water-in-salt electrolytes have gained significant attention due to their advantageous properties [57]. They not only mitigate the effects of H^+^ but also enhance the reversibility of crystal structure transformation during the charge/discharge cycle, thereby improving the cycle life of the battery. The large resistances at the interface between electrolytes and the cathode/anode are the major bottlenecks for delivering desirable electrochemical performances of batteries. The electrolyte/anode interface also suffers from metallic dendrite formation, leading to rapid performance degradation. The use of a solid electrolyte can improve the above problems to a certain extent, with specific methods as follows: (1) surface modification of a solid electrolyte; (2) the application of artificial interlayers; and (3) incorporating multifunctional additives into the electrode material. These methods improve the cycle stability of the battery to a certain extent [58].

With further research, similar limitations of aqueous electrolytes containing salt become apparent. The higher salt concentration increases the viscosity in the cell, limiting transport properties, charge/discharge rates, and performance in advanced zinc batteries. Currently, each electrolyte composition has its advantages and limitations. Selecting the right electrolyte can maximize benefits and result in a battery that meets specific needs across various environments. Therefore, significant progress is still needed in battery research, presenting both opportunities and challenges for future development.

## 5. Conclusions and Outlook

Different types of batteries possess distinct advantages and disadvantages. Multi-ion batteries demonstrate exceptional performance, increased capacity, and extended cycle life; however, they are susceptible to interference from H^+^ ions. Single-ion batteries effectively mitigate the H^+^ issue but may not match the multi-ion batteries in certain aspects. Aqueous battery electrolytes are straightforward to produce, readily available, and widely utilized; nonetheless, they pose specific safety risks. Non-aqueous batteries can bypass these challenges but encounter limitations related to electrolyte composition and cost.

Above, we discussed the effects of different types and a number of roles of ions on battery performance, but in practice, we find that the ionic species in the battery will exceed our expectations for various reasons, and then we need to control the anionic species.

Achieving precise control of phosphate-based polyanion cathodes can be carried out through material synthesis, doping modifications, morphology control, process optimization, and so on. For example, fine synthesis methods such as solvothermal and solid phase sintering are used to ensure the homogeneity and purity of the materials. Precise control of reaction temperature and time can be used to optimize the crystal structure. Doping with other metal ions (e.g., Mg, Al) can be used to improve conductivity and stability. Surface coating of conductive carbon materials can improve electron conduction. Precise control of nanoparticles can be achieved by adjusting reaction conditions (e.g., pH). Optimizing preparation process parameters can improve the specific capacity and cycling stability. Ensuring that the choice of electrolytes is compatible with the cathode material can minimize side reactions.

All our discussions are still relatively biased towards qualitative analysis, and our main subsequent work is to shift the focus of our research towards quantitative analysis, which will be used to determine the optimum composition of phosphate-based polyanion cathodes for practical applications, which can be achieved through design of compositional variables, material characterization, electrochemical testing, and data modeling and analysis. The design of experiments (DOE) method is used to systematically vary the ratio and conditions of different compositions and to evaluate the effect of different doping elements and concentrations on battery properties. X-ray diffraction (XRD) and scanning electron microscopy (SEM) are used to analyze the crystal structure and morphology of the materials, and energy spectrum analysis (EDS) is used to determine the elemental distribution. Cyclic voltammetry (CV) constant current charge/discharge tests are performed to assess specific capacity and cycling stability. Electrochemical impedance spectroscopy (EIS) is used to study the conductivity and charge transfer impedance of materials. The relationship between composition and performance can be modeled using multiple regression analysis or machine learning methods. Key factors affecting performance are identified and optimal composition combinations are predicted. With this systematic approach, the optimal composition of phosphate-based polyanion cathodes can be quantitatively determined to realize their efficient performance in practical applications.

From the above discussion, it is clear that different batteries have their own strengths and limitations, and no single type can meet all usage conditions. The goal now is to enhance existing batteries to better fit various conditions and achieve superior performance. Development and research of aqueous batteries are currently more advanced than those of non-aqueous batteries, both in scale and timeline. Additionally, publications on aqueous batteries have increased at a higher rate compared to non-aqueous batteries. Aqueous batteries, due to their advantages, are likely to remain the dominant choice for the foreseeable future. Therefore, it is crucial to continue analyzing the characteristics of different batteries and determine appropriate research directions moving forward.

**Figure 6:**
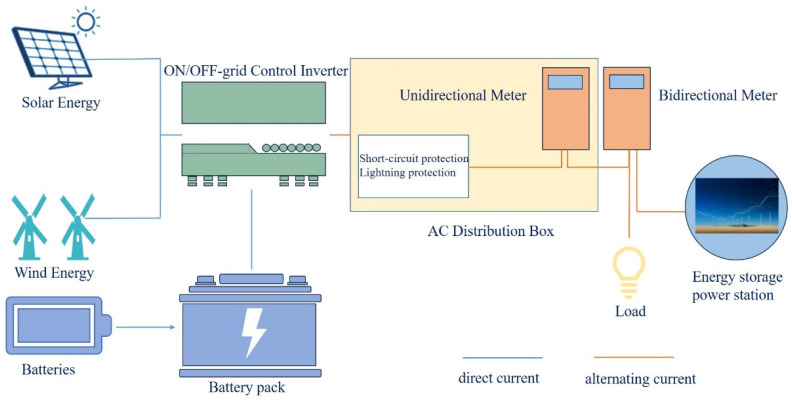
Flow chart of zinc battery to energy storage power station.

As a conduit for energy from production to consumption, batteries play a crucial role in optimizing the allocation of time and space in modern society and have a wide range of applications [59]. Large-scale battery storage systems can be used for grid peaking, improving the utilization of renewable energy, and playing an increasingly important role in the energy sector. Battery energy storage power stations represent an untapped blue ocean. From the perspective of battery structure and power converters, the scalability and modularity of power storage through batteries—coupled with their ability to act as both energy suppliers and consumers—provide significant operational flexibility [60]. Battery energy storage has advantages in scalability, service life, and flexibility. However, current battery energy storage is mainly dominated by technologies such as lithium-ion batteries, liquid flow batteries, lead-acid batteries, and sodium-based batteries. Large-scale applications of battery systems with zinc ions as the anode have yet to be realized. However, low-cost, high-security aqueous zinc-ion batteries have promising prospects for large-scale energy storage [61]. Due to their different working principles and application scenarios, each energy storage technology has its own advantages and limitations. Therefore, physical and chemical energy storage will remain mainstream for quite some time. However, compared with other energy storage methods, electrochemical energy storage has unique advantages. Although zinc-ion energy storage cannot replace higher-power batteries, it still has significant research value. It can be used as a substitute for other batteries in some special circumstances. Under the right conditions, zinc battery energy storage will excel in some aspects compared to other energy storage methods. A simple flowchart illustrating the process from a battery to an energy storage plant is shown in Figure 6. Zinc batteries can be combined to form a battery bank, which, like solar and wind energy, can be converted from direct current (DC) to alternating current (AC) through an integrated off-grid control system. The power can then be utilized comprehensively through an energy storage plant using unidirectional and bidirectional meters in an AC distribution box. This illustrates the simple process from a battery to an energy storage power station, highlighting the potential for large-scale industrial use of zinc battery energy storage, which is a key focus of our future efforts. Therefore, our current focus is to enhance the application scenarios of zinc batteries as conditions allow.

At the same time, we should always pay attention to the application of new technologies in batteries. The future of batteries depends not only on the iterative optimization of existing technologies, but also focuses on the intersection of other disciplines with battery energy storage. Machine learning (ML) and artificial intelligence (AI) are two key areas of computer science. AI and ML have a wide range of applications across a variety of industries, driving automation, personalized service, and efficiency, with tremendous potential for innovation. Optimizing material composition and structure through model predictions to improve conductivity and stability in ML models, including artificial neural networks (ANNs), support-vector machines (SVMs), random forest (RF), partial least squares regression (PLS), and logistic regression (LR), have successfully predicted the properties of battery materials [62]. We can quickly screen a large number of phosphate battery material combinations through these technologies, find high-performance positive and negative electrode and electrolyte materials, and guide the experimental design of phosphate cathode materials. Discovering and exploring untried chemical spaces will help develop new materials with potential applications, and integrate chemistry, physics, and data science to foster multidisciplinary collaboration and improve material quality. These technologies will drive innovation in battery technology and provide more efficient energy storage solutions.

## Figures and Tables

**Table 1:** Comparison of common battery anode elements and their electrochemical properties.

Working Ion	Ionic Radii (Å)	Electrode Potential vs. SHE (V)	Specific Gravimetric Capacity (mAh·g^−1^)	Specific Volumetric Capacity (mAh·g^−1^)
Li^+^	0.76	−3.04	3862	2066
Na^+^	1.02	−2.71	1166	1129
K^+^	1.38	−2.93	685	586
Mg^2+^	0.72	−2.37	2205	3832
Zn^2+^	0.74	−0.76	820	5855
Al^3+^	0.535	−1.66	2980	8046

**Table 2:** Different zinc-ion battery cathode materials and their electrochemical properties.

Cathode	Electrolyte	Voltage/V	Capacity/mAh·g^−1^	Retention%/Cycles	Number (Acting Ions)	Ref.
LiFePO_4_	1 M LiOTf + 1 M Zn (OTf)_2_ + SDBS	0.9–1.4 V	158 (0.5 C)	88.6% at 1 C (100)	3 (Zn^2+^, Li^+^, H^+^)	[20]
LiFePO_4_	4 M Zn(OTf)_2_ + 2 M LiClO_4_	0.9–1.4 V	165 (0.2 C)	90% at 0.2 C (285)	2 (Zn^2+^, H^+^)	[21]
Li_3_V_2_(PO_4_)_3_	1 M Li_2_SO_4_ + 2 M ZnSO_4_	0.7–2.1 V	131 (0.2 C)	85.4% at 0.2 C (200)	3 (Zn^2+^, Li^+^, H^+^)	[22]
Na_3_V_2_(PO_4_)_3_	0.5 M Zn(CH_3_COO)_2_	0.8–1.7 V	92 (0.5 C)	74.0% at 0.5 C (100)	3 (Zn^2+^, Na^+^, H^+^)	[23]
Na_3_V_2_(PO_4_)_3_	2 M Zn(OTf)_2_	0.6–1.8 V	114 (0.05 A·g^−1^)	75.0% at 0.5 A·g^−1^ (200)	3 (Zn^2+^, Na^+^, H^+^)	[24]
Na_3_V_2_(PO_4_)_2_F_3_	2 M Zn(OTf)_2_	0.8–1.9 V	65 (0.08 A·g^−1^)	98.0% at 0.2 A·g^−1^ (600)	2 (Zn^2+^, H^+^)	[25]
Na_3_V_2_(PO_4_)_2_F_3_	3 M Zn(OTf)_2_	0.2–2.0 V	100 (0.2C)	90.0% at 0.2C (600)	2 (Zn^2+^, H^+^)	[26]
VOPO_4_·2H_2_O	21 M LiTFSI + 1 M Zn(Tr)_2_	0.8–2.1 V	139 (0.1 A·g^−1^)	93.0% at 1 A·g^−1^ (1000)	1 (Zn^2+^)	[27]
VOPO_4_·xH_2_O	13 M ZnCl_2_ + 0.8 M H_3_PO_4_	0.7–1.9 V	170 (0.1 A·g^−1^)	91.8% at 2 A·g^−1^ (500)	1 (Zn^2+^)	[28]
VOPO_4_	4 M Zn(OTf)_2_ + 0.5 M Me_3_EtOTf	0.2–1.9 V	163 (0.05 A·g^−1^)	88.7% at 2 A·g^−1^ (6000)	1 (Zn^2+^)	[29]
MgV_2_O_6_·1.7H_2_O	0.1 M Zn(OTf)_2_ in anhydrous acetonitrile + 1% vol water	0.3–1.4 V	425.7 mAh·g^−1^ at 0.2 A·g^−1^	97% at 0.2 A·g^−1^ (50)	2 (Zn^2+^, H^+^)	[30]
